# Ibrutinib sensitizes CLL cells to venetoclax by interrupting TLR9-induced CD40 upregulation and protein translation

**DOI:** 10.1038/s41375-023-01898-w

**Published:** 2023-04-26

**Authors:** Karoline Kielbassa, Marco V. Haselager, Danique J. C. Bax, Bianca F. van Driel, Julie Dubois, Mark-David Levin, Sabina Kersting, Rebecka Svanberg, Carsten U. Niemann, Arnon P. Kater, Eric Eldering

**Affiliations:** 1 Department of Experimental Immunology, Amsterdam UMC location University of Amsterdam, Meibergdreef 9, Amsterdam, the Netherlands; 2 Amsterdam Institute for Infection and Immunity, Cancer Immunology, Amsterdam, the Netherlands; 3 Cancer Center Amsterdam, Cancer Immunology, Amsterdam, the Netherlands; 4 Department of Hematology, Amsterdam UMC location University of Amsterdam, Meibergdreef 9, Amsterdam, the Netherlands; 5 Department of Internal Medicine, Albert Schweitzer Hospital, Dordrecht, the Netherlands; 6 Haga Teaching Hospital, Den Haag, the Netherlands; 7 Department of Hematology, Rigshospitalet, Copenhagen, Denmark; 8 Department of Clinical Medicine, University of Copenhagen, Copenhagen, Denmark

**Keywords:** Apoptosis, Cancer microenvironment, Cell signalling

## Abstract

Chronic lymphocytic leukemia (CLL) cells upregulate Bcl-2 proteins within the lymph node (LN) microenvironment. Signaling via B-cell receptor, Toll-like receptors and CD40 collectively reduce sensitivity to the BCL-2 inhibitor venetoclax. Time-limited treatment with venetoclax plus the BTK-inhibitor ibrutinib results in deep remissions, but how this combination affects LN-related signaling is not yet completely clear. Therefore, samples obtained from the HOVON141/VISION phase 2 clinical trial were used to analyze this. Two cycles of lead-in ibrutinib monotherapy resulted in decreased protein expression of Bcl-2 proteins in circulating CLL cells. Strikingly, at this timepoint CD40-induced venetoclax resistance was strongly attenuated, as was expression of CD40. Since CD40 signaling occurs within the CLL LN, we tested various LN-related signals that could affect CD40 signaling. While BCR stimulation had only a minor effect, TLR9 stimulation via CpG led to significantly increased CD40 expression and importantly, reverted the effects of ibrutinib treatment on venetoclax sensitivity by inducing overall protein translation. Together, these findings identify a novel effect of ibrutinib: interruption of TLR9-induced CD40 upregulation and translation of pro-survival proteins. This mechanism may potentially further inhibit priming of CLL cells in the LN microenvironment for venetoclax resistance.

## Introduction

Within the lymph node (LN) microenvironment, CLL cells are exposed to interactions with non-malignant immune cells leading to inhibition of apoptosis and induction of proliferation [1]. In particular, CD40-CD40L interactions between CLL cells and follicular T-helper cells in the LN promote NF-κB and mTOR activation, resulting in CLL survival and drug resistance by upregulation of anti-apoptotic Bcl-2 family members Bcl-XL, Mcl-1 and Bfl-1 [2–5]. Various studies have supported that CD40 signaling is involved in CLL proliferation and provides a model for tumor microenvironment (TME)-induced drug resistance [2, 5–8]. A successful therapeutic strategy in CLL is to induce apoptosis directly by so-called BH3 mimetics. The Bcl-2-specific inhibitor venetoclax is highly cytotoxic for CLL cells and causes a rapid reduction in circulating CLL cells in the majority of patients, but LN responses are less complete [9]. Another successful therapy for CLL is the use of ibrutinib, which targets the B-cell receptor (BCR) signaling pathway [10]. Ibrutinib treatment results in efflux of CLL cells from the proliferative lymphoid tissue into the peripheral blood (PB) [11, 12], thereby preventing CLL cells from receiving microenvironmental survival signals and in that way halting disease progression [13, 14]. However, neither of these therapies are curative as single treatment agents and repeated or continuous treatment is required, thereby increasing the risk of developing resistance to therapy and disease progression [15]. The combination of ibrutinib and venetoclax may have synergistic anti-tumor effects, since ibrutinib forces CLL cells from LNs to the PB where they become fully dependent on Bcl-2, and thus vulnerable to venetoclax [16]. Phase 2 and 3 outcomes of time-limited ibrutinib plus venetoclax combination trials have demonstrated high response rates when used as first-line therapy and in relapsed/refractory CLL [17–22].

In a previous study, we investigated the role of ibrutinib on the expression of Bcl-2 family members and drug resistance in a small set of randomly obtained clinical samples. We found that CLL cells that recently left the LN had higher Bcl-XL and Mcl-1 expression compared to cells immigrating back to the LN. This distinction in expression collapsed upon ibrutinib treatment yet the pretreatment profile reappeared upon relapse [23]. In order to investigate whether changes in expression levels of Bcl-2 family proteins might correlate with clinical response to venetoclax-containing regimens, we studied PB samples of CLL patients after two cycles of ibrutinib in the context of the HOVON141/VISION clinical trial [21]. In this study, two 28-day cycles of ibrutinib lead-in is followed by MRD-guided combination treatment of venetoclax and ibrutinib. We analyzed changes in Bcl-2 protein levels at the end of cycle 2 of ibrutinib monotherapy in relation to MRD status obtained at cycle 9 (after 7 cycles of ibrutinib + venetoclax combination) [18, 21, 24]. We observed that two cycles of ibrutinib treatment resulted in a collapse of CD40-mediated venetoclax resistance. This correlated well with previous suggestions that ibrutinib might, besides BCR signaling, also affect CD40 signaling, at least in vitro [25, 26]. Whether this can also occur in vivo [27], either directly or indirectly, is as yet unknown which led us to investigate mechanistically how ibrutinib affects CD40 signaling. Since Bcl-2 family proteins are induced in the LN as a result of microenvironment-induced signaling, we hypothesized that ibrutinib treatment may interrupt those in vivo signals due to relocalization of CLL cells to the PB. Consequently, we investigated which signals present in the LN microenvironment may explain how ibrutinib affects TME-mediated resistance to venetoclax.

## Materials and methods

### Patient material

PB mononuclear cells (PBMCs) were collected and cryopreserved from relapsed or refractory CLL patients enrolled in the phase 2 clinical trial performed by the Dutch-Belgian Cooperative Trial Group for Hematology Oncology (HOVON): the HOVON141/VISION trial (ClinicalTrials.gov, NTC03226301). In addition, paired LN and PBMC patient material, as well as PBMC from treatment naïve CLL patients were obtained through the B-cell malignancies Biobank at Amsterdam UMC. The patient characteristics are depicted in Supplementary table 1. The study was approved by the medical ethics committee at the Amsterdam UMC. Written informed consent from all subjects was obtained in accordance with the Declaration of Helsinki. Primary CLL samples outside of Hovon141 clinical trial that were included in this study contained 88–99% CD5^+^/CD19^+^ cells.

### Study design

HOVON141/VISION enrolled 225 patients with a creatinine clearance ≥30 mL/min with previously treated CLL, with or without TP53 aberrations requiring treatment according to International Workshop on Chronic Lymphocytic Leukemia (iwCLL) 2018 [28]. The treatment regimen consisted of ibrutinib monotherapy (420 mg days 1–28; cycle 1 and 2), followed by combined venetoclax (ramp up to 400 mg) and ibrutinib (420 mg; cycles 3–15). Participants with undetectable MRD (<10^−4^; less than one chronic lymphocytic leukemia cell per 10.000 leukocytes) [24] were randomly assigned (1:2) to ibrutinib maintenance or treatment cessation. Patients who were MRD positive continued to receive ibrutinib monotherapy. A full protocol description has been published previously [29].

### Cell culture and detection of apoptosis

Lymphocytes of CLL patients were co-cultured with NIH3T3 fibroblasts stably transfected with human CD40L or negative control as described before [30, 31]. 3T40 fibroblasts were STR profiled and tested as mycoplasma-negative. After 24 h, CLL cells were detached and incubated with or without drugs for an additional 1, 2, 4 or 24 h. CLL cell viability was measured by flow cytometry using DiOC6 and TO-PRO-3 viability dyes. For BCR stimulation, either soluble anti-IgM or anti-IgM-coated dynabeads (Invitrogen, Waltham, Massachusetts, USA) were used. All used reagents are listed in Supplementary table 2.

### Flow cytometry

Single-cell suspensions were stained with the following antibodies: anti-CD3 (BD Biosciences, #BD561416), CD19 (BD Biosciences, #BD555412), CD184/CXCR4 (Biolegend, #306514), CD5 (Biolegend, #300619), CD40 (Beckman Coulter, #IM1936U) and CD95 (BD Biosciences, # 555673). Cells were subsequently permeabilized and stained with the following antibodies: anti-Bcl-2 (Biolegend, #658709), Bcl-XL (Cell Signaling Technology, #13835S), Mcl-1 (Cell Signaling Technology, #65617S), anti-A1/Bfl-1 was a kind gift of Prof. Dr. J. Borst (Leiden University Medical School, The Netherlands) and p-S6 (Cell Signaling Technology, #5364). Unconjugated antibodies were stained with secondary goat anti-rabbit IgM+IgG antibody (Southern Biotech, #4010-09 S). Stained cells were analyzed on a FACS Canto II cytometer (BD Biosciences, San Jose, CA, USA). Expression levels of surface markers CD5 and the chemokine receptor CXCR4 were used to distinguish between CLL cells that recently left lymphoid tissue (LN emigrants; CD5high, CXCR4dim) from older cells that re-enter LN tissue (LN immigrants; CD5dim, CXCR4high) [23, 32]. Within FlowJo™ v10 Software (BD Biosciences, San Jose, CA, USA), we applied an algorithm that reproducibly detects 9 quadrants of the total CLL population to distinguish the LN immgrants from the LN emigrants in an unbiased fashion (Supplementary Fig. 1A). Analysis of the LN emigrant population before and after two cycles of ibrutinib indicated a significant reduction of CD5 after ibrutinib treatment, while CXCR4 expression was significantly increased, suggesting that CLL cells are skewed towards a LN immigrant phenotype compared to baseline (Supplementary Fig. 1B, C). In the case of Bcl-2 family member expression, gMFIs were normalized by setting the baseline LN immigrant population at 100%, and subsequently plotting only data for the emigrant population, as described in Haselager et al. [23].

### Western blot analysis and DNA-binding ELISA

Western blot analysis was performed using standard techniques. Membranes were probed with the following antibodies: anti-p100/p52 (Cell Signaling Technology, #4882), p-p65 (Cell Signaling Technology, #3033), p-S6 (Cell Signaling Technology, #5364), actin (Santa Cruz Biotechnology, # sc-1616), Bim (StressMarq, #SPC-113D). Odyssey Imager (Li-Cor Biosciences) was used as a detection method according to the manufacturer’s protocol. Nuclear extracts of CLL cells were prepared using NE-PER kit (ThermoFisher, Waltham, Massachusetts, USA). Transcription factor activity of p52 and p65 was accessed using TransAM NF-κB Family Kit (Active Motif, Carlsbad, United States).

### Real-time polymerase chain reaction

Total RNA was isolated from primary CLL cells using the RNeasy Mini Kit (Qiagen) and cDNA was synthesized by reverse transcriptase reactions according to the manufacturer’s instructions (Promega). Products were amplified in a Fast SYBR green (Life Technologies) reaction (40 cycles of 5 s at 95 °C followed by 30 s at 60 °C). Primers used: CD40-Forward: TGATGTTGTCTGTGGTCCCC. CD40-Reverse: GGCAAACAGGATCCCGAAG. BCL-2-Forward: ATGTGTGTGGAGAGCGTCAA. BCL-2-Reverse: CAGTTCCACAAAGGCATCCCAG. BCL2L1-Forward (Bcl-XL): GTATTGGTGAGTCGGATCGC. BCL2L1-Reverse (Bcl-XL): TGCTGCATTGTTCCCATAGA.

### Protein synthesis assay

Global protein synthesis was measured using the Click-iT Plus OPP Protein Synthesis Assay Kit (ThermoFisher) and following protocol of Yeomans et al. (2016) [33]. *O*-propargyl-puromycin (OPP; 20 μM) was added to 0.5 × 10^6^ cells and incubated for 30 min. Cells were fixed and permeabilized using the Cytofix/Cytoperm Fixation Permeabilization Kit (BD Biosciences, San Jose, CA, USA). Alexa-Fluor-488 was conjugated to OPP as described in the manufacturer’s instructions and cells were stained with anti-CD19 (BD Biosciences, #BD555412) and CD5 (Biolegend, #300619). Cells were measured on a FACS Canto II flow cytometer (BD Biosciences) and analyzed using FlowJo v10.8. As a control, cells were treated with cycloheximide for 5 min before OPP addition and fluorescence of cycloheximide-treated cells was subtracted from all experimental values.

### Statistics

To ensure adequate power of statistical testing, sample sizes were chosen based on the type of material used, including at least two primary LN samples and three primary CLL cells/clinical trial samples. Additional patient samples were included to ensure that variation within each group of data was similar. For every figure, we applied the appropriate statistical test. Normality tests were applied to ensure normal data distribution. The paired sample *t*-test was used to analyze paired observations. Two-way ANOVA test was used to analyze differences between groups. *p < 0.05; **p < 0.01; ***p < 0.001; ****p < 0.0001.

## Results

### Ibrutinib treatment causes collapse of Bcl-2 family member expression which does not correlate with mid-treatment MRD response with the combination

**Fig. 1:**
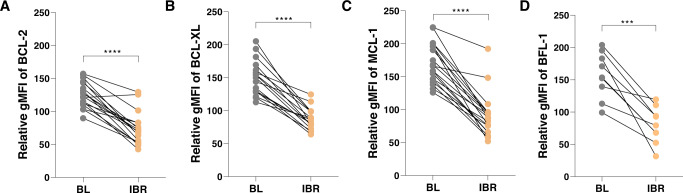
Ibrutinib treatment causes collapse of Bcl-2 family member expression. **A**–**D** Immunological detection of Bcl-2 family members in LN emigrants before and after Ibrutinib treatment (*N* = 17; BFL-1 *N* = 9). gMFIs were normalized by setting the baseline LN immigrant population at 100%, and by subsequently plotting only the emigrant population. Two-Way ANOVA test was used for statistical analyses.

Previously we reported an upregulation of pro-survival Bcl-2 family members in the LN of patients with CLL [6, 34], which correlated with increased expression of Bcl-XL and Mcl-1 in the LN ‘emigrant’ versus ‘immigrant’ fraction of circulating CLL cells, defined by opposing expression levels of CD5 and CXCR4. This differences collapsed upon ibrutinib treatment [23]. Here, we applied an algorithm to reproducibly select LN immigrant and emigrant populations, by automated gating of 9 quadrants (Supplementary Fig. 1A). Application of this method in a large cohort of samples from the HOVON141 clinical trial validated differential Bcl-2 member expression in these two circulating leukemia fractions. Furthermore, we now included Bfl-1 expression in the analysis. For Bcl-XL, Mcl-1 and Bfl-1, we found increased levels in CLL cells emigrating from the LN. After two months of ibrutinib treatment, a significant reduction of the pro-survival proteins Bcl-2, Bcl-XL, Mcl-1 and Bfl-1 was observed (*p* < 0.0001 for Bcl-2, Bcl-XL and Mcl-1 and *p* < 0.001 for Bfl-1) (Fig. 1A–D).

To investigate whether the relative changes in protein expression correlated with clinical responses, we divided patients based on positive or undetectable MRD at the end of treatment cycle 9 (after 7 cycles of combination) [24] or based on IGHV mutational status. After analysis of a subset of patients (*N* = 17, *N* = 9 for Bfl-1), the changes in expression levels of Bcl-2 family members did not correlate with mid-treatment MRD status or IGHV mutational status. Of note, also the baseline levels in these proteins did not correlate with subsequent MRD or IGHV status (Supplementary Fig. 2), implying that the expression levels of Bcl-2 family members do not serve as early biomakers for later clinical responses.

### Ibrutinib treatment attenuates CD40-mediated venetoclax resistance

**Fig. 2:**
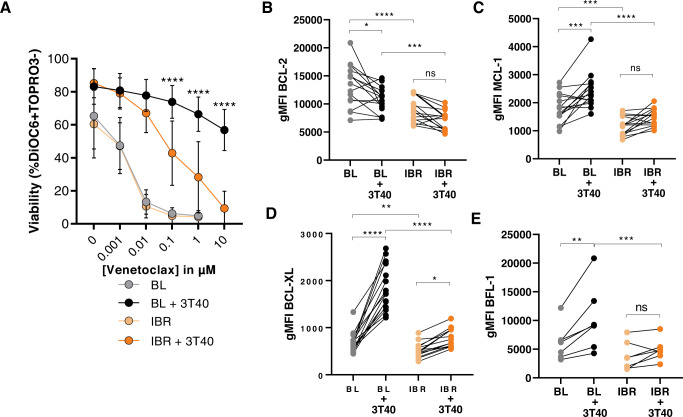
Ibrutinib treatment attenuates CD40-mediated venetoclax resistance. **A** Patient samples obtained at baseline and after two months ibrutinib treatment were unstimulated or cultured on CD40L-expressing 3T3 fibroblasts (3T40), followed by in vitro venetoclax treatment. Viability data were measured by flow cytometry using DiOC6/TO-PRO-3 staining (*N* = 17). **B**–**E** Immunological detection of Bcl-2 family members before and after in vitro CD40 stimulation (3T40) in patient samples at baseline and after ibrutinib treatment (*N* = 15; BFL-1 *N* = 7). Two-way ANOVA test was used for statistical analyses.

We next studied possible consequences of ibrutinib-mediated shifts in the expression of Bcl-2 members on venetoclax sensitivity. Despite reduction in Bcl-2 family protein expression upon ibrutinib treatment, in vitro treatment with venetoclax for 24 h showed no differences in venetoclax sensitivity between ex vivo PB samples collected at baseline and after two months of ibrutinib treatment (Fig. 2A). We applied co-cultures of CLL cells with CD40L-expressing 3T3 fibroblasts (3T40L) to mimic TME-induced venetoclax resistance. As shown before, samples from untreated patients demonstrated almost complete venetoclax resistance upon CD40 stimulation [6]. Strikingly, two cycles of ibrutinib treatment strongly attenuated in vitro CD40-induced resistance to venetoclax (to 100-fold increase in IC50; *p* < 0.0001; Fig. 2A). This was parallelled by a significantly higher induction of Bcl-XL, Mcl-1 and Bfl-1 expression upon in vitro CD40 activation in baseline samples compared to ibrutinib-treated samples (*p* < 0.0001) (Fig. 2C–E). Together, these data demonstrate that CD40-mediated venetoclax resistance was reduced after ibrutinib treatment.

### Downstream signaling via CD40 is unaffected by two cycles of ibrutinib treatment

**Fig. 3:**
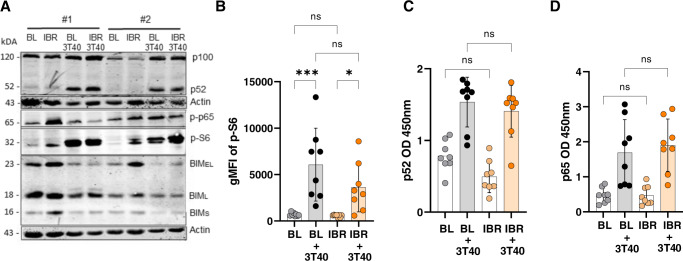
Downstream signaling via CD40 is unaffected after ibrutinib treatment. **A** Western blot of peripheral blood collected from two patients. CLL cells obtained at baseline (BL) and after two months of ibrutinib treatment (IBR) were unstimulated or co-cultured on CD40L-expressing fibroblasts (3T40) for 24 h. Protein lysates were probed for NF-kB proteins (p100, p52 and p-p65), pS6, pro-apoptic Bim and actin as loading control. More patients are included in Supplementary Fig. 3. **B** Immunological detection of phospho-S6 expression before and after in vitro CD40 stimulation in patient samples obtained at baseline and after ibrutinib treatment (*N* = 7). **C**, **D** DNA-binding ELISA of NF-κB family members showed that CD40-mediated NF-kB activity of p52 and p65 was not affected after two months ibrutinib treatment (*N* = 8). Two-Way ANOVA test was used for statistical analyses.

We next investigated how ibrutinib affects signaling downstream of CD40. CD40 triggering activates downstream Akt/mTOR, NF-κB and Erk signaling in CLL cells [13, 35, 36]. We first tested effects of ibrutinib treatment on NF-κB signaling as this pathway is responsible for induction of Bcl-XL and Bfl-1 that contribute strongly to venetoclax resistance [2, 23]. Using read-outs for canonical (p65) and non-canonical (p52) NF-κB activity, we could not detect significant reduction in NF-κB activity after two cycles of ibrutinib (Fig. 3A, Supplementary Fig. 3). The same was true for pS6, a marker of Akt-mTOR activity, both by Western blot and by flow cytometry (Fig. 3A, B). Pro-apoptotic Bim was increased after ibrutinib treatment, and reduced after CD40/Erk activation both as previously described [6, 37] (Fig. 3A, Supplementary Fig. 3). Moreover, CD40-induced NF-κB activity as measured by p65 and p52 DNA-binding activity seemed unaffected after two months ibrutinib treatment (Fig. 3C, D). These data strongly suggest that although CD40 protein expression, upregulation of Bcl-2 proteins, and induction of venetoclax resistance were reduced after ibrutinib treatment, CD40 downstream signaling remained intact.

### In vitro ibrutinib treatment shows no direct effects on CD40-induced venetoclax resistance or Bcl-2 family expression

**Fig. 4:**
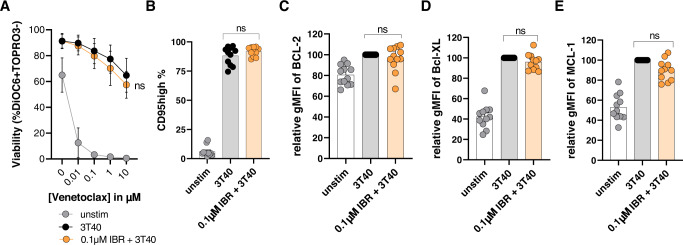
In vitro ibrutinib treatment shows no direct effects on CD40-induced venetoclax resistance or Bcl-2 family member expression. In vitro experiments using 0.1 µM ibrutinib for 24 h showed no direct effects of ibrutinib on **A** venetoclax sensitivity (*N* = 14), **B** immunological detection of CD95 (*N* = 11) and (**C–E**) and Bcl-2 family proteins (*N* = 12) as seen in vivo. Paired sample *t*-test was used for statistical analyses.

To establish whether ibrutinib directly affects CD40 signaling, we performed in vitro experiments in which CLL cells were pretreated with 0.1 µM ibrutinib for 24 h followed by co-culture with 3T3 or 3T40L fibroblasts for another 24 h. CD40-activated CLL cells that were treated with ibrutinib in vitro did not show a significant reduction in venetoclax resistance compared to the 3T40L control (Fig. 4A). Furthermore, expression levels of the activation marker CD95 were unaffected upon 0.1 µM ibrutinib treatment (Fig. 4B). Finally, the expression levels of Bcl-2, Bcl-XL and Mcl-1 after CD40 activation also showed no significant differences upon in vitro ibrutinib treatment (Fig. 4C–E). Higher concentrations of 1 µM ibrutinib were also tested and showed similar results (Supplementary Fig. 4). These data indicate no direct effects of ibrutinib on CD40-mediated venetoclax resistance, suggesting that the effect of in vivo ibrutinib treatment on CD40 signaling in CLL cells happens indirectly.

### CD40 expression is increased in the lymph node emigrants mediated by TLR9 signaling

**Fig. 5:**
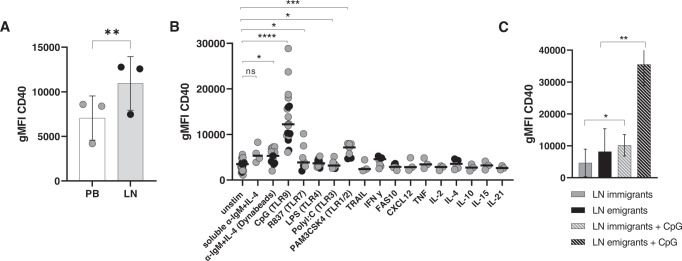
CD40 expression is increased in the lymph node emigrants mediated by TLR9 signaling. **A** Immunological detection of CD40 in lymph node (LN) single-cell suspension compared to its paired peripheral blood sample (PB) confirmed increased CD40 expression in the LN (*N* = 3). **B** Different in vitro stimulations were used to identify which signals are involved in the upregulation of CD40 expression. Stimuli were added to CLL cells for 24 h and expression levels of CD40 were analyzed by flow cytometry. Soluble αIgM + IL-4 (*N* = 4), αIgM-coated beads + IL-4 (*N* = 11), CpG (*N* = 18), TLR4 (*N* = 10), TLR1/2, TLR3 and TLR7 (*N* = 7), IL-4 and FAS10 (*N* = 6), TRAL, CXCL12, TNF, IL-2/10/15/21 (*N* = 3). Black symbols represent IGHV mutated CLL patients (*N* = 6), while gray symbols represent IGHV unmutated CLL patients (*N* = 12). **C** Combined staining of CXCR4 and CD5 within the CLL population showed increased CD40 expression in the LN emigrant (CD5highCXCR4dim) subsets compared to LN immigrants (CD5dimCXCR4high) upon 24 h CpG (1 µg/ml) stimulation (*N* = 3). Paired sample *t*-test was used for statistical analyses.

Since in vivo ibrutinib treatment prevented CD40-mediated upregulation of Bcl-2 family proteins whereas downstream CD40 signaling was unaffected, we next studied expression levels of CD40. Analysis of several paired LN and PB samples indicated that CD40 expression on CLL cells is upregulated in the LN (Fig. 5A). Therefore, we next investigated which in vivo signals could be involved in the upregulation of CD40 in the LN niche. We tested various in vitro stimuli to mimic pathway activation as observed in LN-derived CLL cells in vivo. Of more than 10 candidates tested, only TLR1/2,3,7 and especially TLR9 stimulation via PAM3CSK4, Poly:IC, R837 and CpG respectively, induced CD40 expression significantly in both IGHV mutated and unmutated CLL (*p* < 0.0001). In comparison, BCR stimulation via α-IgM-coated dynabeads plus IL-4 as a known strong surrogate for LN signals [38] showed only a minor but still significant increase in CD40 expression in CLL cells (*p* ≤ 0.05)(Fig. 5B). Analysis of CD40 expression upon stimulation with CpG revealed increased CD40 expression specifically in the LN emigrant population (*p* < 0.001)(Fig. 5C). CpG stimulation did not skew CLL LN emigrant/immigrant phenotypes themselves (Supplementary Fig. 5). Therefore, these data strongly suggest a TLR9-mediated effect on CD40 expression in CLL cells that recently left the LN.

### Ibrutinib affects CD40 expression in a non-transcriptional manner

**Fig. 6:**
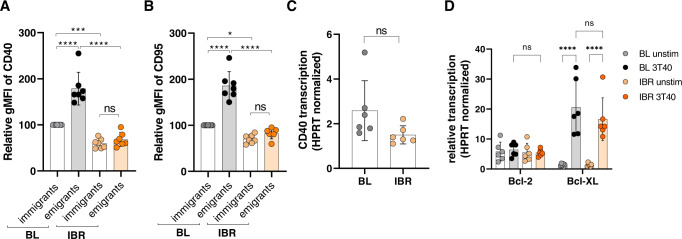
Ibrutinib affects CD40 protein expression in a non-transcriptional manner. **A**, **B** Immunological detection of CD40 and CD95 in LN emigrants (CD5highCXCR4dim) compared to LN immigrants (CXCR4highCD5dim) of patient samples obtained at baseline and after two months ibrutinib treatment (*N* = 7). **C** CD40 mRNA expression levels were measured by real-time PCR in patient samples obtained at baseline and after two months ibrutinib treatment (*N* = 7). **D** Bcl-2 family member mRNA expression levels were measured by real-time PCR. Samples were normalized to HPRT (*N* = 6). Paired sample *t*-test was used for statistical analyses.

We next studied the effects of ibrutinib treatment on the expression levels of CD40 and its activation marker CD95 in LN immigrants and emigrants according to the automated gating strategy as described above (Supplementary Fig. 1A). Consistent with what we observed in LN-derived CLL cells, at baseline LN emigrants showed significantly increased CD40 and CD95 protein expression levels compared to LN immigrants (*p* < 0.0001) (Fig. 6A, B). After ibrutinib treatment, CD40 and CD95 expression levels were significantly reduced in both LN immigrant and emigrant populations, indicating that in vivo ibrutinib treatment leads to impaired CD40 expression and activation (*N* = 7, *p* < 0.001). Although CD40 protein expression was reduced after ibrutinib treatment, transcription of *CD40* was unaffected compared to baseline (Fig. 6C). Furthermore, transcriptional induction of the Bcl-2 family members *Bcl-2* and *Bcl-XL* was also maintained after ibrutinib treatment (Fig. 6D), suggesting that in vivo ibrutinib treatment affects CD40 signaling in a post-transcriptional manner.

### TLR9 stimulation promotes protein translation, thereby reverting the effects of ibrutinib on CD40-mediated venetoclax resistance

**Fig. 7:**
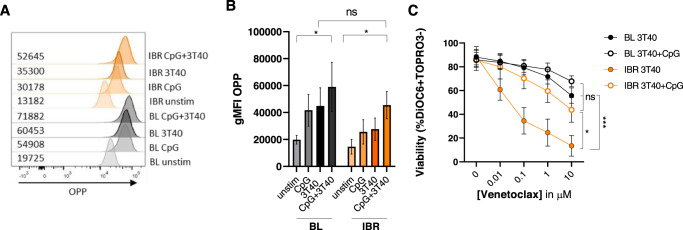
TLR9 stimulation promotes protein translation, thereby reverting the effects of ibrutinib on CD40-mediated venetoclax resistance. **A**, **B** Protein synthesis assay using O-propargyl-puromycin (OPP), which is incorporated into newly translated proteins and fluorescently labeled (*N* = 3). As a control, fluorescence of cycloheximide-treated cells was subtracted from all experimental values. Paired sample *t*-test was used for statistical analyses. **C** Baseline and ibrutinib-treated CLL cells were stimulated with CpG (1 µg/ml) and cultured on 3T40L for 24 h. After detachment, cells were treated with venetoclax for an additional 24 h. Viability data was measured by flow cytometry using DiOC6/TO-PRO-3 staining (*N* = 5). Paired sample *t*-test was used for statistical analyses.

Effects of ibrutinib treatment on translation were studied by measuring global protein synthesis. Either TLR9 or CD40 stimulation could induce protein translation to a certain extent, yet the combination of TLR9 and CD40 stimulation induced protein translation even further (Fig. 7A, B). Protein translation was reduced upon ibrutinib treatment, though the combination of TLR9 and CD40 stimulation was able to induce protein translation in ibrutinib-treated samples back to the baseline 3T40 control. Finally, we probed the role of TLR9 stimulation on CD40-induced venetoclax resistance, before and after in vivo ibrutinib treatment. In CD40-stimulated CLL cells of ibrutinib-treated patients, TLR9 stimulation via CpG led to increased venetoclax resistance (*p* < 0.05), almost to the same extent as observed at baseline (*N* = 5) (Fig. 7C). These data are consistent with our results on protein translation and indicate that TLR9 stimulation is able to revert ibrutinib-mediated effects on CD40-mediated venetoclax resistance. In summary, these findings demonstrate that TLR9 signaling may play a role in CLL drug resistance in the LN microenvironment by promoting protein translation, thereby increasing susceptibility of CLL cells to CD40-mediated pro-survival signaling.

## Discussion

Most evidence for driving CLL proliferation and disease progression is attributed to the CLL LN microenvironment, where CLL cells receive various signals activating downstream signaling pathways [1, 13]. BCR signaling is a major driver in vivo, and there is also compelling evidence that CD40 and additional signaling is involved in CLL proliferation and drug resistance [2, 3, 6, 7, 13, 39–41]. Therefore, it is relevant to understand which signals, present in the LN microenvironment, regulate CD40 expression and what effect ibrutinib treatment has on CD40 signaling. Our combined data indicate an unrecognized interplay between TLR and CD40 signals in determining potential LN-associated resistance to venetoclax, which is interrupted already during early stages of ibrutinib treatment. This mechanistic insight extends previous transcriptional data on reduced expression of TLR- and CD40-driven gene sets under ibrutinib [27]. We confirmed induction of pro-apoptic Bim [37] and a collapse of all anti-apoptotic Bcl-2 family members after ibrutinib treatment [23], yet venetoclax sensitivity was not increased ex vivo. Also shorter incubations with venetoclax did not show differences in venetoclax sensitivity after ibrutinib treatment compared to baseline (Supplementary Fig. 6). A possible explanation might be that the difference in the expression of Bcl-XL, Mcl-1 and Bfl-1 between unstimulated baseline and post-ibrutinib treatment emigrant cells is much smaller compared to the difference in expression of these anti-apoptotic proteins in the same cells following CD40 activation. Moreover, PB samples obtained after 1 week of combination treatment with ibrutinib and venetoclax showed an upregulation of Bcl-2 and Mcl-1 as seen previously for single venetoclax treatment [23], while Bcl-XL expression remained reduced (Supplementary Fig. 7). With respect to potential utility as biomarker for later clinical responses, we tested whether the expression level changes differed between prognostic groups, or correlated with later MRD responses. This appeared not to be the case, though it cannot be excluded that by studying larger patient numbers, or possibly relapse samples, such predictive aspects might be found. Furthermore, the lack of correlation between expression of these proteins in emigrant cells and clinical responses might be due to higher levels of these proteins in actual LN cells compared to the emigrant CLL subpopulation. Previously we reported that soluble CD40L was not sufficient to induce venetoclax resistance in vitro [42]. We therefore use a co-culture system of CLL cells with 3T40 fibroblasts, as a model for TME-induced venetoclax resistance. Though distinct from gradually emerging venetoclax resistance in relapsing patients as seen in the clinic, the underlying mechanisms may have a common basis. A key finding was the reduction of in vitro CD40-induced venetoclax resistance upon two cycles of ibrutinib treatment compared to baseline. Downstream CD40 signaling and trancription, and also NF-κB activity were unaffected after two cycles of ibrutinib treatment. A previous study reported decreased NF-κB transcription factor activity measured by p50 on day 28 of ibrutinib treatment compared to pretreatment in CLL cells [43]. As this was in unstimulated CLL cells, it actually fits well with our data in CD40-stimulated cells, together emphasizing that cells in PB are devoid of NF-κB stimulatory signals, yet retain capacity to respond to them. Instead of direct attenuation of CD40 downstream signaling capacity, we found that ibrutinib treatment affects overall protein translation which includes expression levels of CD40 receptor. This is in agreement with previous studies showing that protein expression of the oncogene MYC is increased in the LN and is reduced after ibrutinib treatment [33, 44]. In addition, a previous study demonstrated that in the LN microenvironment, expression of miR29, which targets the positive regulator of CD40 signaling TRAF4, is suppressed through BCR-induced activation of MYC [45]. It was suggested that BCR/MYC effects on miR29 correlated with IGHV mutation status [45]. Since our data did not show such a correlation (Supplementary Fig. 2), this would indicate that the BCR-MYC-miR29-TRAF4 axis is distinct from the link between TLR and CD40 pathways described here.

Our data implies that under ibrutinib treatment, if cells cannot (re-)enter LN sites, a factor is lacking that maintains or induces CD40 expression. We showed that TLR9 stimulation via CpG led to increased CD40 expression in CLL cells specifically in the LN emigrant population. These findings fit well with increased TLR pathway activity in LN-derived CLL cells as identified by gene array studies [41, 46], as well as in situ proximity ligation assays reporting interactions of pIκBa with TLR9 and MyD88 in LN-derived CLL cells [46]. This is consistent with our finding that TLR9 expression was increased in CLL LN emigrants (Supplementary Fig. 8). Another previous study demonstrated the formation of a multiprotein supercomplex composed of MYD88, TLR9, and the BCR (My-T-BCR) as a mode of oncogenic BCR signaling in various lymphomas, yet this was not evident in CLL LN biopsies [47]. This suggests that CLL may depend on an alternative type of BCR-enhancing signaling, and does not exclude a complex involving pIkBa and TLR9 [46]. Considering that there is no direct proof that CLL cells receive signals through the TLR9 pathway in vivo, a recent study proposed a role for mitochondrial DNA (mtDNA) that harbors hypomethylated CpG motifs similar to bacterial DNA in the in vivo activation of TLR9 signaling [48]. A potential mechanism to trigger TLR9 in vivo might be via mtDNA released into the cytosol via BAX/BAK-mediated mitochondrial outer membrane pores followed by extrusion and permeabilisation of the inner mitochondrial membrane [49], providing ligands for triggering cGAS/STING or TLRs. Furthermore, mtDNA might be also transmitted via exosomes or directly via mitochondrial transfer between cells in the TME [50]. Interestingly, a contrasting notion was presented very recently by Martines et al. who demonstrated that in mouse models of CLL/Richter syndrome, TLR/IRAK4 mediated signaling did not play an intrinsic role in propagation of the malignant cells [51]. Formally, the available data are not mutually exclusive, as TLR involvement in human CLL LN in situ might be more prominent or required than in the aggressive mouse TCL1 and Richter mouse models. In vitro CpG stimulation induces phosphorylation of CD79A, LYN and SYK, implying a potential overlap between TLR and BCR signaling [13]. However, there is a strong correlation between CpG-mediated CLL proliferation and IGHV mutation status which may suggest that TLR activity is regulated by BCR signaling [52, 53]. Other studies demonstrated an important role for TLR signaling in CLL pathogenesis and maintaining CLL cell viability during ibrutinib therapy, suggesting superior anti-tumor activity by combining ibrutinib with drugs targeting TLR signaling [46, 48]. Targeting TLR signaling might affect the CLL cells that remain in the LNs during ibrutinib treatment and in non-responsive patients, thereby inhibiting protein translation and consequently preventing a resistant CLL phenotype induced by microenvironmental signals.

In conclusion, ibrutinib treatment broadly affects Bcl-2 family protein expression and interrupts a triad of signaling pathways involving BCR, CD40 and TLR9. Our combined data indicate a novel aspect of ibrutinib efficacy, specifically its potential capacity to interrupt TLR9-induced CD40 upregulation and protein translation, which normally primes CLL cells in the LN environment for venetoclax resistance. With respect to implications for potential combination scenarios, our findings suggest that simultaneous administration of ibrutinib and venetoclax might be more beneficial than sequential single treatment.

## Supplementary information

Supplemental Material

## Data Availability

All data are available from the corresponding author on reasonable request.
