# Supplementary material for: Ibrutinib sensitizes CLL cells to venetoclax by interrupting TLR9-induced CD40 upregulation and protein translation
Source: Leukemia. 2023 Apr 26;37(6):1268–76. doi: 10.1038/s41375-023-01898-w (PMC10244160; doi:10.1038/s41375-023-01898-w)
Supplement: Supplementary file 1 — Supplemental Material [file 41375_2023_1898_MOESM1_ESM.pdf]

Supplemental Table 1. Patient characteristics

Patient ID	timepoint	study	sample date	treatment	age	sex	IGHV mutation status	ALC ($\times10^9$/L)	%lymphocytes	%CD5CD19	FISH	Article Figure
H1410178 (#1)	baseline	HOVON141	11-10-2018	-	75	F	mutated	42.85	95.3	96.29	13q14 deletion	1A-C, 2A-E, 3A+C-D, 5C, 6A-D, 7C
H1410178 (#1)	2 months	HOVON141	6-12-2018	ibrutinib	75	F	mutated	173.1	97.3	98.94	13q14 deletion	1A-C, 2A-E, 3A+C-D, 5C, 6A-D, 7C
H1410169 (#2)	baseline	HOVON141	19-9-2018	-	73	M	mutated	32.41	88.5	92.68	11q deletion	1A-C, 2A-E, 3A, 5C, 6A-D, 7C
H1410169 (#2)	2 months	HOVON141	14-11-2018	ibrutinib	73	M	mutated	104.07	95.6	96.35	11q deletion	1A-C, 2A-E, 3A, 5C, 6A-D, 7C
H1410003 (#3)	baseline	HOVON141	26-7-2017	-	69	M	mutated	201.68	94.6	98.12	13q14, 17p13 deletion	1A-C, 2A-E, 3B, 6A-C
H1410003 (#3)	2 months	HOVON141	3-10-2017	ibrutinib	69	M	mutated	360	NA	98.06	13q14, 17p13 deletion	1A-C, 2A-E, 3B, 6A-C
H1410092 (#4)	baseline	HOVON141	23-5-2018	-	57	F	mutated	62.18	98	95.21	13q14 deletion	1A-C, 2A-E, 3B-D, 6A-B
H1410092 (#4)	2 months	HOVON141	18-7-2018	ibrutinib	57	F	mutated	265.27	98.5	97.67	13q14 deletion	1A-C, 2A-E, 3B-D, 6A-B
H1410009	baseline	HOVON141	5-9-2017	-	52	F	unmutated	238.6	95	90.63	13q14 deletion	1A-C, 2A-D, 3C-D
H1410009	2 months	HOVON141	31-10-2017	ibrutinib	52	F	unmutated	360	NA	92.38	13q14 deletion	1A-C, 2A-D, 3C-D
H1410010	baseline	HOVON141	5-9-2017	-	58	F	unmutated	248.9	97	97.07	13q14 deletion	1A-C, 2A-D, 7C
H1410010	2 months	HOVON141	31-10-2017	ibrutinib	58	F	unmutated	306	NA	97.44	13q14 deletion	1A-C, 2A-D, 7C
H1410011	baseline	HOVON141	12-9-2017	-	60	M	unmutated	56	162.8	98.61	13q14 deletion	1A-D, 2A-D, 3B-D
H1410011	2 months	HOVON141	8-11-2017	ibrutinib	60	M	unmutated	359.5	96.6	99.12	13q14 deletion	1A-D, 2A-D, 3B-D
H1410027	baseline	HOVON141	21-11-2017	-	59	F	unmutated	170.7	89.6	70.49	13q14, 17p13 deletion	1A-D, 2A-D, 3B-D, 6D
H1410027	2 months	HOVON141	16-1-2018	ibrutinib	59	F	unmutated	99.56	86.9	75.47	13q14, 17p13 deletion	1A-D, 2A-D, 3B-D, 6D
H1410045	baseline	HOVON141	9-1-2018	-	42	M	unmutated	65.36	89.3	84.18	trisomy 12, 13q14 deletion	1A-D, 2A-D, 6D
H1410045	2 months	HOVON141	7-3-2018	ibrutinib	42	M	unmutated	69.39	89.4	90.9	trisomy 12, 13q14 deletion	1A-D, 2A-D, 6D
H1410055	baseline	HOVON141	1-2-2018	-	66	F	unmutated	218.07	93.3	91.75	13q14 deletion	1A-C, 2A, 3B-D, 7B-C
H1410055	2 months	HOVON141	29-3-2018	ibrutinib	66	F	unmutated	235	97	99.37	13q14 deletion	1A-C, 2A, 3B-D, 7B-C
H1410059	baseline	HOVON141	6-2-2018	-	64	F	mutated	128.22	95.7	96.75	13q14 deletion	1A-D, 2A-E, 3B-D
H1410059	2 months	HOVON141	5-4-2018	ibrutinib	64	F	mutated	162.8	97.9	96.17	13q14 deletion	1A-D, 2A-E, 3B-D
H1410068	baseline	HOVON141	13-3-2018	-	72	M	unmutated	19.72	60.6	90.94	none	1A-C, 2A-D
H1410068	2 months	HOVON141	8-5-2018	ibrutinib	72	M	unmutated	21.82	74.9	95.96	none	1A-C, 2A-D
H1410074	baseline	HOVON141	13-3-2018	-	62	M	unmutated	60.17	85.7	97.56	13q14, 17p13 deletion	1A-C, 2A
H1410074	2 months	HOVON141	23-5-2018	ibrutinib	62	M	unmutated	486.7	96.6	92.97	13q14, 17p13 deletion	1A-C, 2A
H1410082	baseline	HOVON141	13-4-2018	-	65	M	mutated	29.91	74.6	75.96	13q14 deletion	1A-C, 2A-D
H1410082	2 months	HOVON141	8-6-2018	ibrutinib	65	M	mutated	75.79	92.4	90.69	13q14 deletion	1A-C, 2A-D
H1410095	baseline	HOVON141	16-5-2018	-	71	M	mutated	126.55	92.5	98.02	13q14 deletion	1A-C, 2A-E, 3B-D, 6A-C, 7B
H1410095	2 months	HOVON141	6-7-2018	ibrutinib	71	M	mutated	157	95.7	95.79	13q14 deletion	1A-C, 2A-E, 3B-D, 6A-C, 7B
H1410116	baseline	HOVON141	7-6-2018	-	74	M	unmutated	211.21	93	98.78	11q, 13q14, 17p13 deletion	1A-D, 2A-E, 3B, 5C, 6A-D, 7B-C
H1410116	2 months	HOVON141	1-8-2018	ibrutinib	74	M	unmutated	224.93	97.6	99.68	11q, 13q14, 17p13 deletion	1A-D, 2A-E, 3B, 5C, 6A-D, 7B-C
H1410167	baseline	HOVON141	14-9-2018	-	60	M	unmutated	42.76	88.4	95.51	13q14 deletion	1A-D, 2E, 6D
H1410167	2 months	HOVON141	20-11-2018	ibrutinib	60	M	unmutated	144.02	98.7	98.98	13q14 deletion	1A-D, 2E, 6D
H1410182	baseline	HOVON141	24-10-2018	-	77	M	mutated	68.8	91.6	91.1	none	1A-D
H1410182	2 months	HOVON141	20-12-2018	ibrutinib	77	M	mutated	228.26	97.8	96.06	none	1A-D
H1410183	baseline	HOVON141	23-10-2018	-	75	F	mutated	349.27	97	99.09	13q14 deletion	1A-D, 2A-E,3B, 6A-C
H1410183	2 months	HOVON141	21-12-2018	ibrutinib	75	F	mutated	420.2	97.2	99.07	13q14 deletion	1A-D, 2A-E,3B, 6A-C
H1390012 PBMC	baseline	HOVON139	27-3-2017		69	M	unmutated	76.4	77	NA	11q, 13q14 deletion	5A
H1390012 LN	baseline	HOVON139	27-3-2017		69	M	unmutated	76.4	77	NA	11q, 13q14 deletion	5A
H1390036 PBMC	baseline	HOVON139	1-11-2017		62	M	unmutated	NA	NA	NA		5A (used in two independent experiments)
H1390036 LN	baseline	HOVON139	1-11-2017		62	M	unmutated	NA	NA	NA		5A (used in two independent experiments)
1826	-	-	10-10-2016	-	85	F	unmutated	300.23	NA	97.7		4A,C-E
1935	-	-	8-5-2017	-	52	F	mutated	242.7	94	96.17	13q14 deletion	4A,C-E
1759	-	-	9-5-2016	-	63	M	unmutated	250.91	96.3	97.6	11q deletion	4A,C-E
1760	-	-	9-5-2016	-	51	F	mutated	122.75	NA	92.4		4A,C-E
1214	-	-	30-5-2012	-	52	M	unmutated	48.4	72	91.1	13q14 deletion	4A-B
1895	-	-	14-2-2017	-	65	F	mutated	112.5	NA	97	trisomy 12, 13q14 deletion	4A,C-E
1799	-	-	2-9-2016	-	67	M	unmutated	333.7	99	96.8		4A-B
1355	-	-	21-5-2013	-	71	F	mutated	44.8	90.5	95.3		4A-B
2379	-	-	2-10-2018	-	76	M	unmutated	111.51	92.9	94.88	13q14 deletion	5B
356	-	-	6-3-2001	-	75	M	unmutated	NA	NA	96.18		5B
2747	-	-	14-11-2019	-	63	M	unmutated	51.07	84.2	96.47		4A-B, 5B
2229	-	-	24-5-2018		62	M	unmutated	51.01	87.8	95.55		5B
2679	-	-	10-9-2019	-	77	M	unmutated	160.19	86.8	95.29	13q14 deletion	5B
898	-	-	1-2-2010	-	75	M	unmutated	127	NA	93	13q14 deletion	5B
2088	-	-	16-1-2018	-	83	M	unmutated	72.08	59.4	88.54		5B
2148	-	-	19-3-2018	-	81	M	mutated	111.97	94.6	97.07		5B
2373	-	-	27-9-2018	-	84	F	mutated	52.76	86.9	94.96		5B
2454	-	-	11-12-2018	-	78	F	mutated	92.14	88.4	96.25	13q14 deletion	5B
2591	-	-	12-6-2019	-	63	M	unmutated	41.31	86.2	94.39		5B
1983	-	-	17-7-2017	-	38	F	unmutated	46.69	83.4	92.34		4A,5B
2363	-	-	17-9-2018	-	75	F	mutated	292.68	96.8	98.48	trisomy 12, 13q14 deletion	4A,5B
1988	-	-	25-7-2017	-	45	F	mutated	181.53	91.9	96.28	13q14 deletion	4A,5B
1871	-	-	12-1-2017	-	64	F	NA	96.8	72.9	94,98		4A
2346	-	-	3-9-2018	-	65	F	NA	162.82	95.3	96.34		4A
2906	-	-	24-2-2020	-	60	M	unmutated	97.79	88.8	97.79	trisomy12, 11q, 13q14 deletion	5B
2594	-	-	17-6-2019	-	39	M	unmutated	261.47	97.1	97.96	trisomy12, 11q, 13q14 deletion	5B
3612	-	-	30-9-2021	-	67	M	unmutated	61.72	85.3	93.8		5B
3100	-	-	20-7-2020	-	70	F	mutated	99.05	94.3	97.76	11q deletion	5B

ALC = Absolute leukocyte count, F = female, M = male, NA = not available.

Supplemental Table 2. Reagents

Reagent	Supplier	Concentration
Ibrutinib for Hovon141 clinical trial	Janssen (Beerse, Belgium)	
Ibrutinib for in vitro experiments	Selleckchem (Texas, USA)	0.1 µM
Venetoclax	Active Biochem (Bonn, Germany)	0.001-10 µM
ODN2006 (Class B CpG oligonucleotide)	InvivoGen (Toulouse, France)	1µg/ml
goat F(ab) α-human IgM	Sanbio (Uden, The Netherlands)	20 µg/ml
PAM3CSK4	InvivoGen (Toulouse, France)	10 µg/ml
Poly-I:C	InvivoGen (Toulouse, France)	10 µg/ml
LPS	Merck (Darmstadt, Germany)	1 µg/ml
R837	InvivoGen (Toulouse, France)	5 µg/ml
IFN-γ	Bio-Techne (Minneapolis, USA)	50 ng/ml
human IL-2	PeproTech (London, UK)	50 ng/ml
human IL-4	Thermo Fisher Scientific (Waltham, USA)	25 ng/ml
human IL-10	Bio-Techne (Minneapolis, USA)	50 ng/ml
human IL-15	PeproTech (London, UK)	50 ng/ml
human IL-21	Thermo Fisher Scientific (Waltham, USA)	50 ng/ml
human superkiller TRAIL	Enzo LifeSciences (Bruxelles, Belgium)	50 ng/ml
CXCL12	PeproTech (London, UK)	200 ng/ml
TNFα	R&D systems (Minneapolis, USA)	50 ng/ml

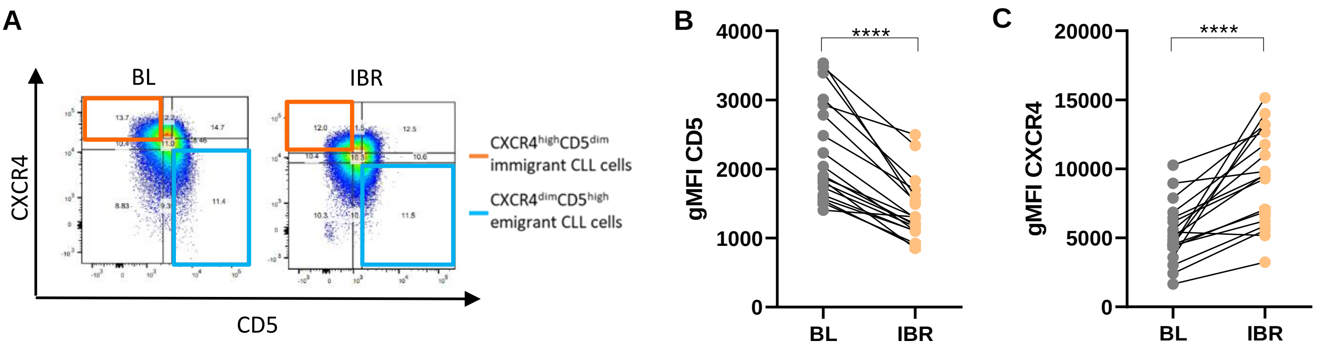

Supplemental Figure 1. Ibrutinib treatment changes CLL cells towards a LN immigrant phenotype
(A) Combined staining of CXCR4 and CD5 within the CLL population allows discrimination of LN emigrants (CD5highCXCR4dim) from LN immigrants (CXCR4highCD5dim) at baseline (BL) and after two months ibrutinib treatment (IBR). For all following experiments, an algorithm was applied to divide the total CLL population into 9 quadrants in an unbiased fashion. (B-C) Flow cytometry analysis of the expression levels of CD5 and the chemokine receptor CXCR4 (N=19) before and after ibrutinib treatment. TWO-Way Anova test was used for statistical analyses.

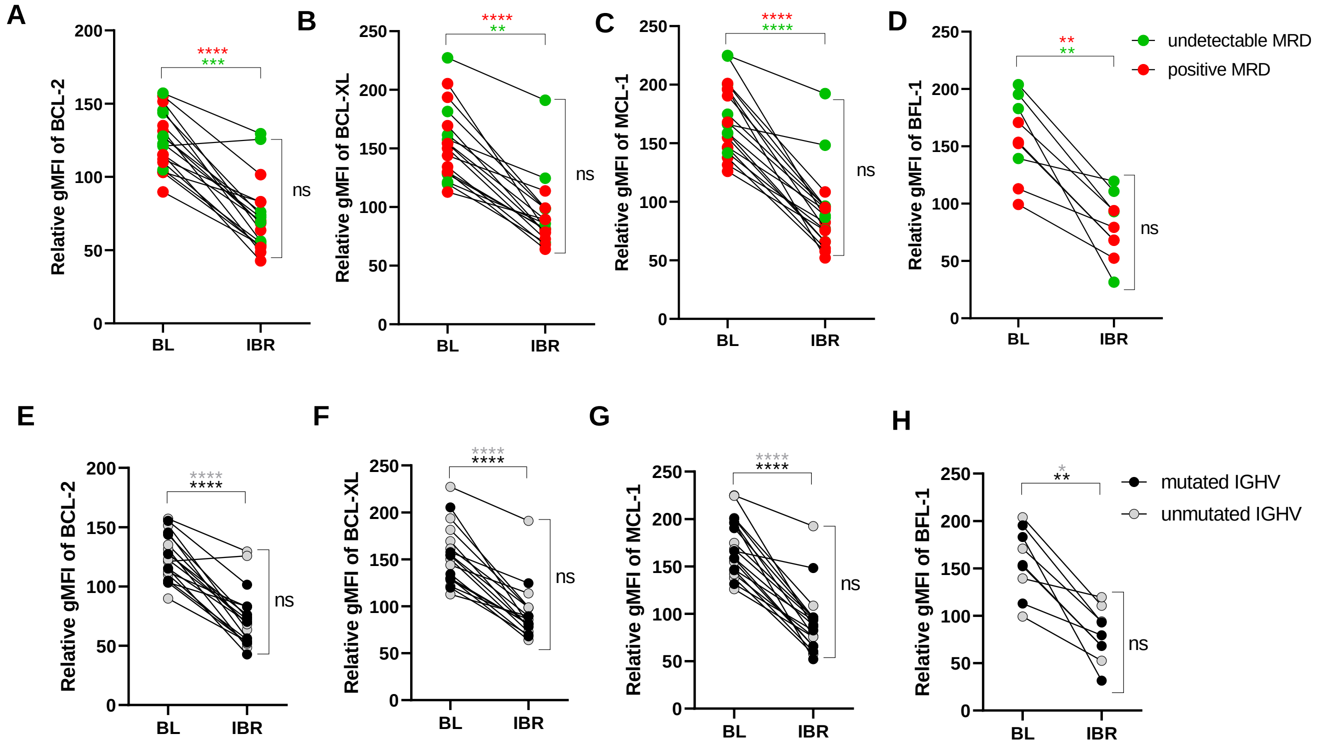

Supplemental Figure 2. Ibrutinib-mediated collapse of Bcl-2 family member expression does not correlate with mid-treatment MRD response with the combination

Immunological detection of Bcl-2 family members in LN emigrants before and after ibrutinib treatment (N=17; BFL-1 N=9). Data is divided based on positive MRD or undetectable MRD obtained at end cycle 9 of the trial (A-D) or IGHV mutational status (E-H), respectively.

gMFIs were normalized by setting the baseline LN immigrant population at 100%, and by subsequently plotting only the emigrant population. TWO-Way Anova test was used for statistical analyses.

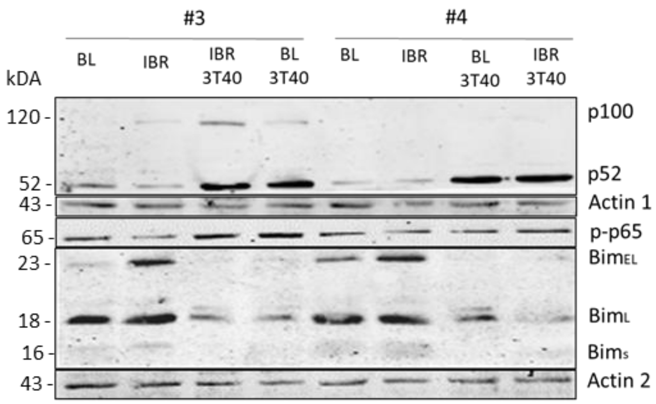

Supplemental Figure 3. Downstream mediators of CD40 are unaffected after ibrutinib treatment

(A) Western blot of peripheral blood collected from 2 patients. CLL cells obtained at baseline (BL) and after two months of ibrutinib treatment (IBR) were unstimulated or co-cultured on CD40L expressing fibroblasts (3T40) for 24h. Protein lysates were probed for NF-kB proteins (p100, p52 and pp65), pro-apoptic Bim and actin as loading control.

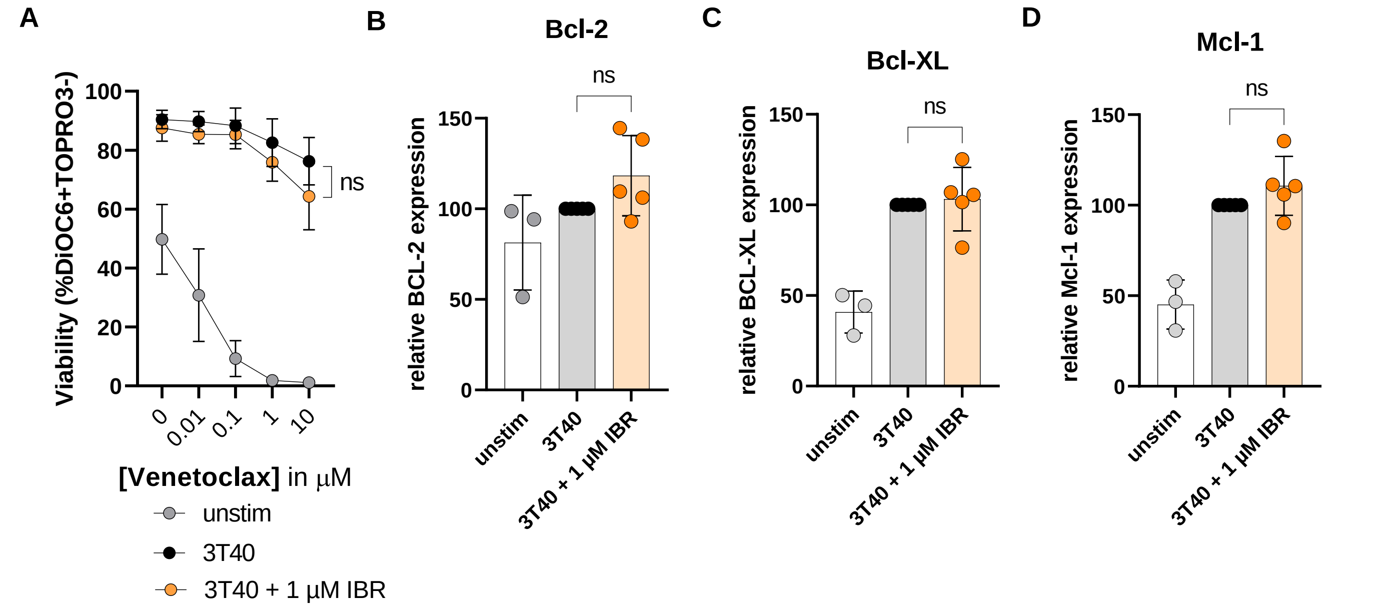

Supplemental Figure 4. *In vitro* experiments revealed no direct ibrutinib-mediated effects on CD40-induced venetoclax resistance

In vitro experiments using 1 µM ibrutinib for 24h showed no direct effects of ibrutinib on (A) venetoclax sensitivity and (B-D) Bcl-2 family proteins (N=5) as seen *in vivo*. Paired sample t-test was used for statistical analyses.

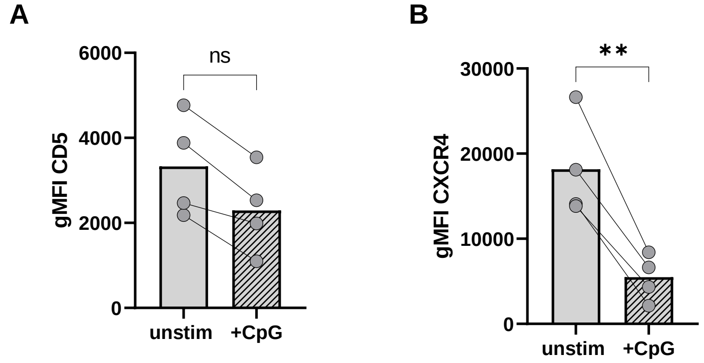

Supplemental Figure 5. Immunological detection of CD5 (A) and CXCR4 (B) in total CLL population before and after CpG stimulation (1µg/ml ODN2006)(N=4). Paired t-test was used for statistical analyses.

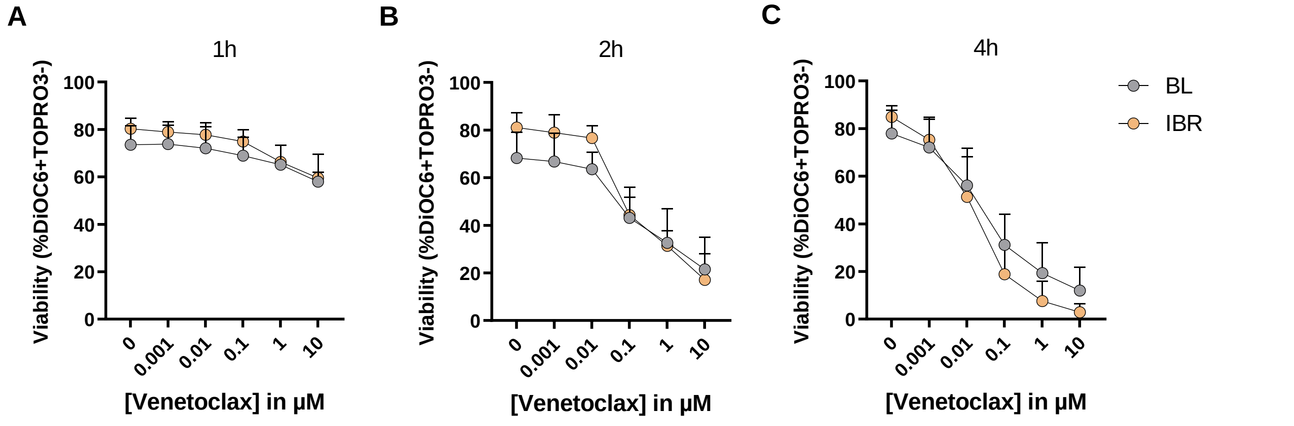

Supplemental Figure 6. Shorter *in vitro* venetoclax treatment does not affect venetoclax sensitivity after *in vivo* ibrutinib treatment

Ex vivo patients CLL cells obtained at baseline (BL) and after two months ibrutinib treatment (IBR) were treated with venetoclax *in vitro* for A) 1 hour (N=4), B) 2 hours (N=4) or C) 4 hours (N=6). Viability data was measured by flow cytometry using DiOC6/TO-PRO-3 staining.

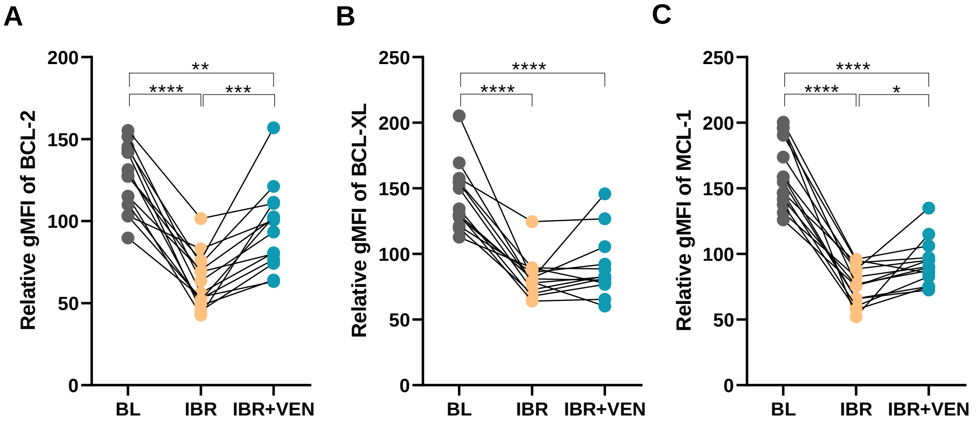

Supplemental Figure 7. Bcl-2 family members after single ibrutinib treatment and combined venetoclax and ibrutinib treatment

(A-C) Immunological detection of Bcl-2 family members in LN emigrants before, after single ibrutinib treatment and after combined treatment with venetoclax and ibrutinib (N=14). GMFIs were normalized by setting the baseline LN immigrant population at 100%, and by subsequently plotting only the emigrant population. TWO-Way ANOVA test was used for statistical analyses.

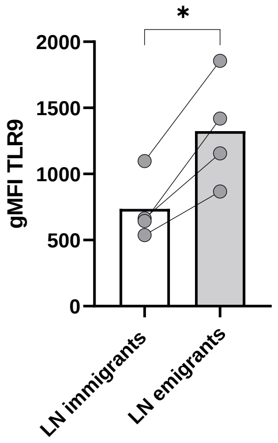

Supplemental Figure 8. Immunological detection of TLR9 in LN emigrants (CD5highCXCR4dim) and LN immigrants (CXCR4highCD5dim)(N=4). Paired t-test was used for statistical analyses.
